# An Inotropic Action Caused by Muscarinic Receptor Subtype 3 in Canine Cardiac Purkinje Fibers

**DOI:** 10.1155/2013/207671

**Published:** 2013-10-23

**Authors:** Katsuharu Tsuchida, Yumiko Mizukawa, Tetsuro Urushidani, Shigehiro Tachibana, Yukiko Naito

**Affiliations:** ^1^Department of Rational Medicinal Science, Faculty of Pharmaceutical Sciences, Doshisha Women's College, Kyoto, Kyotanabe-shi 610-0395, Japan; ^2^Department of Pathological Physiology, Faculty of Pharmaceutical Sciences, Doshisha Women's College, Kyoto, Kyotanabe-shi 610-0395, Japan; ^3^Department of Pharmacology, Hatano Research Institute, Food and Drug Safety Center, Kanagawa, Hadano-shi 257-8523, Japan

## Abstract

*Objective*. The objective of this study was to investigate the inotropic mechanisms and the related muscarinic receptor subtype of acetylcholine (ACh) in canine cardiac Purkinje fibers. *Materials and Methods*. Isolated Purkinje fiber bundles were used for the measurement of contraction. The receptor subtype was determined using PCR and real-time PCR methods. *Results*. ACh evoked a biphasic response with a transient negative inotropic effect followed by a positive inotropic effect in a concentration-dependent manner. The biphasic inotropic actions of ACh were inhibited by the pretreatment with atropine. Caffeine inhibited the positive inotropic effect of ACh. ACh increased inositol-1,4,5-trisphosphate content in the Purkinje fibers, which was abolished by atropine. Muscarinic subtypes 2 (M_2_) and 3 (M_3_) mRNAs were detected in the canine Purkinje fibers albeit the amount of M_3_ mRNA was smaller than M_2_ mRNA. M_1_ mRNA was not detected. *Conclusion*. These results suggest that the positive inotropic action of ACh may be mediated by the activation of IP_3_ receptors through the stimulation of M_3_ receptors in the canine cardiac Purkinje fibers.

## 1. Introduction

Five muscarinic acetylcholine receptors (mAChRs) have been identified and studied extensively and functionally elucidated [1]. Functions of these ACh receptor subtypes have been determined in the heart as well as other tissues or organs [2]. A lot of experiments have showed that M_2_ of mAChRs plays an important role in exerting the negative chronotropic action in the sinoatrial node or the negative inotropic action in the atria by activating potassium channels and/or inhibiting adenylate cyclase via G_i/o_ protein. It has been demonstrated that the stimulation of ACh exerts the biphasic inotropic actions in the atria and ventricles of various species [2]. The negative inotropy is attributable to the activation of G_i/o_ in atrial muscles, whereas the mechanisms of ACh-induced positive inotropic action are assumed to be due to the activation of G_q_ protein in the ventricles and atria [2–4]. However, in cardiac Purkinje fibers, the existence or function of muscarinic receptor subtypes still remains a little bit uncertain compared with atria and ventricles. There have been several mechanical tensions, electrophysiological, or biochemical studies regarding the effects of ACh on cardiac Purkinje fibers [5–8]. Curiously enough in the cardiac Purkinje fibers, there has however been no obvious study drawing the conclusion that the stimulation of muscarinic 3 receptors causes the ACh-evoked positive inotropy. In this study, we examined the underlying mechanisms by which ACh evokes the positive inotropic response in the canine Purkinje fibers.

## 2. Materials and Methods

### 2.1. Preparation for the Bundles of Fibers and Experimental Protocols of Contractile Force Measurements

Twelve mongrel dogs (13–18 kg, both sexes), purchased from Kitayama Labes (Nagano, Japan), were used. The animals were intravenously anesthetized with sodium pentobarbital (30 mg/kg). Their hearts were quickly removed by lateral thoracotomy. Purkinje fibers were excised from the ventricles in cold oxygenated Tyrode's solution. As already reported in our previous papers [9], fibers less than 0.8 mm in a diameter and 6–10 mm in length were immersed in a tissue bath (20 mL in volume) with the following composition (mM): NaCl 140.3, KCl 2.7, CaCl_2_ 1.8, MgCl_2_ 1.1, NaH_2_PO_4_ 0.4, NaHCO_3_ 11.9, and glucose 5.6. The solution was adjusted to pH 7.4 by NaOH and was gassed with 95% O_2_ and 5% CO_2_; the temperature of the bath was maintained at 36-37°C during the tension experiment. The bundles of fibers were stimulated at a rate of 1 Hz, with rectangular pulses of 2–4 ms duration and at a voltage of twice the threshold voltage through a bipolar platinum electrode attached to the preparation and connected to an electric stimulator (SEN-7103, Nihon Kohden, Tokyo, Japan). Prior to beginning the study regarding the developed tension, a 60–90 min equilibration was allowed. During equilibration, Tyrode's solution was replaced with fresh solution every 20–30 min. Developed tension was measured with an isometric force-displacement transducer (SB-1T, Nihon Kohden) with the application of a 0.5 g preload.

### 2.2. IP_3_ Production in Canine Cardiac Purkinje Fibers Stimulated with Acetylcholine

Five male beagle dogs weighing 11–14 kg (Covance Research Products, Kalamazoo, MI, USA) were used. According to the above mentioned method, Purkinje fibers were excised from the ventricles and immersed in a mini-Magnus tube (2 mL in volume, Medical Kishimoto, Kyoto, Japan) filled with Tyrode's solution. This solution was gassed with 95% O_2_/5% CO_2_, and its temperature was maintained at 36°C (pH 7.4). After equilibration, Purkinje fibers were incubated with ACh 10^−4^ M for 90 sec. To examine the inhibitory effect of muscarinic receptors on IP_3_ production, pretreatment with atropine 10^−5^ M for 3 min was performed before the incubation with ACh. The fibers incubated in Tyrode's solution without these reagents were regarded as control. These reagents (20 *μ*L) were added directly to a 2 mL mini-Magnus containing Tyrode's solution. After the incubation, Purkinje fibers were frozen immediately in liquid nitrogen and stored at −80°C until measurements of IP_3_. The methods of measurements of IP_3_ levels were as follows. Frozen Purkinje fibers were homogenized in ice-cold 10 v/v% perchloric acid with Ultra Sonic Homogenizer (UH-50, SMT, Tokyo, Japan). After centrifugation at 2,000 ×g for 10 min at 4°C, the supernatants obtained were neutralized to pH 7.5 with ice-cold 1.5 M potassium hydroxide solution containing 60 mM HEPES and centrifuged at 2,000 ×g for 10 min at 4°C. The supernatants were used for a subsequent measurement of IP_3_ concentration as samples. IP_3_ concentration was determined by Inositol-1,4,5-trisphosphate [^3^H] Radioreceptor Assay Kit (PerkinElmer Life & Analytical Sciences, Waltham, MA, USA) according to the instruction manual. Assay was carried out in duplicate. Samples and radiolabeled IP_3_ tracer (^3^H) were mixed and incubated for 1 h on ice. After centrifugation at 2,500 ×g for 15 min at 4°C, the pellets were collected and dissolved in 0.15 M sodium hydroxide solution. Pellets dissolved were kept statically at room temperature for 10 min. They were poured into the glass vials and mixed with Atomlight scintillation cocktail (PerkinElmer Life & Analytical Sciences). Radioactivity (^3^H) was determined by liquid scintillation counter (LS-3801, Beckman Coulter, Fullerton, CA, USA). In addition, protein concentration in the samples was determined according to the Bradford method (Bio-Rad Laboratories, Hercules, CA, USA) using bovine serum albumin (Sigma-Aldrich, St. Louis, MO, USA) for standard curve. Absorbance was detected by the microplate reader (Varioskan Flash, Thermo Fisher Scientific, Waltham, MA, USA).

### 2.3. Reverse Transcription-Polymerase Chain Reaction (RT-PCR)

Five other male beagle dogs (Covance Research Products, Kalamazoo, MI, USA) were used to collect Purkinje fibers. Dissected Purkinje fibers (60–130 mg) from three dogs were soaked in the tubes containing 1 mL of RNAlater (Life Technologies, Carlsbad, CA, USA). Dissected atrial muscles and salivary glands (60–185 mg) were also collected likewise as positive controls. The samples were kept at 4°C for overnight and then stored at −80°C until use. Total RNA was isolated using RNeasy mini kit (Qiagen, Hilden, Germany). The samples were homogenized in 1.0–1.4 mL of Buffer RLT (a component of the kit) with zirconium beads using a Mixer Mill MM301 (Retsch, Haan, Germany). An aliquot of the tissue homogenate (350 or 700 *μ*L) was mixed with 2 volumes of TRIzol LS reagent (Life Technologies, Carlsbad, CA, USA) and an equivalent volume of chloroform. The mixture was centrifuged at 18,000 ×g for 15 min at 4°C, and the resultant aqueous layer was transferred to a new tube. An equivalent volume of 70% ethanol was added to the solution and mixed by pipetting. The mixture was transferred to an RNeasy mini column. The following procedure was performed according to the manufacturer's instructions. In order to remove genomic DNA, on-column digestion by DNase I was performed during the procedure. The concentration of the total RNA was determined by absorbance at 260 nm. Purity and integrity of the total RNA were confirmed by absorbance ratio at 260 and 280 nm and agarose gel electrophoresis. One microgram of total RNA from each sample was reverse-transcribed using QuantiTect Reverse Transcription kit (Qiagen, Hilden, Germany) according to the manufacturer's instructions. A control sample was prepared in parallel with each cDNA sample by replacing reverse transcriptase with RNase-free water. One-fortieth of each reverse transcription (RT) product was used for the following polymerase chain reaction (PCR), which was conducted using Taq DNA polymerase (Takara Bio, Otsu, Japan) and primers for canine M_1_, M_2_, or M_3_ receptors of mAChRs. The primers were purchased from Qiagen (Cf_CHRM1_1_SG, Cf_CHRM2_2_SG, or Cf_CHRM3_1_SG QuantiTect Primer Assay). Cycling conditions were as follows: initial denaturation (4°C for 1 min, 94°C for 1 min), followed by 28 cycles of denaturation (96°C for 20 sec), annealing (60°C for 30 sec), and extension (72°C for 30 sec). An additional extension step (72°C for 7 min) was performed. The RT-PCR products were separated and visualized in ethidium bromide containing 3% agarose gel by electrophoresis.

One-hundredth of each RT product was used for quantitative real-time PCR. Real-time PCR was performed with Rotor-Gene Q (Qiagen, Hilden, Germany) using SYBR premix Ex Taq II (Takara Bio, Otsu, Japan) with the following thermal profile: initial denaturation step (95°C for 3 min), 40 cycles of denaturation (95°C for 10 sec), annealing (60°C for 10 sec), extension step (72°C for 30 sec), and final step for melting curve generation (65–95°C, 1°C per sec). Primers were from Qiagen (Cf_RPLP0_2_SG QuantiTect Primer Assay, or primers for mAChRs used also for semi-quantitative RT-PCR). The cycle threshold (Ct) values were measured. The amount of each gene was calculated using standard curve obtained from serially diluted PCR amplicon which was cloned into pCRII-TOPO vector (Life Technologies, Carlsbad, CA, USA) and linearized by digestion with XhoI. Relative expression levels of mAChR subtypes were calculated as ratios of target amounts to the amount of a housekeeping gene, “ribosomal protein, large P0 (RPLP0)”.

All the animals used in the present experiments were kept in air-conditioned room and supplied with solid foods (DS-A, Oriental Yeast, Tokyo, Japan) and water freely. Animal experiments were conducted in compliance with “Guideline for Animal Experiment in Hatano Research Institute, Food and Drug Safety Center, Taisho Pharmaceuticals, or Doshisha Women's College.”

### 2.4. Chemicals

The following drugs were used: acetylcholine chloride (Sigma-Aldrich, St. Louis, MO, USA); atropine sulfate monohydrate (Wako Pure Chemical Industries, Osaka, Japan); l-isoproterenol hydrochloride (Nikken Chemicals, Tokyo, Japan); caffeine sodium benzoate (Sigma-Aldrich). The agents were dissolved in distilled water as stock solution. On the day of experiment, fresh solution was made from stock by adding to external recording solution.

### 2.5. Statistical Analysis

Statistical significance tests were performed by repeated measures of one-way ANOVA, paired Student's *t*-test, or Wilcoxon's *t*-test. The values were expressed as the mean ± SEM. *P* value of less than 0.05 was considered statistically significant. 

## 3. Results

### 3.1. Characteristic Changes of Contractile Force Evoked by Acetylcholine

**Figure 1:**
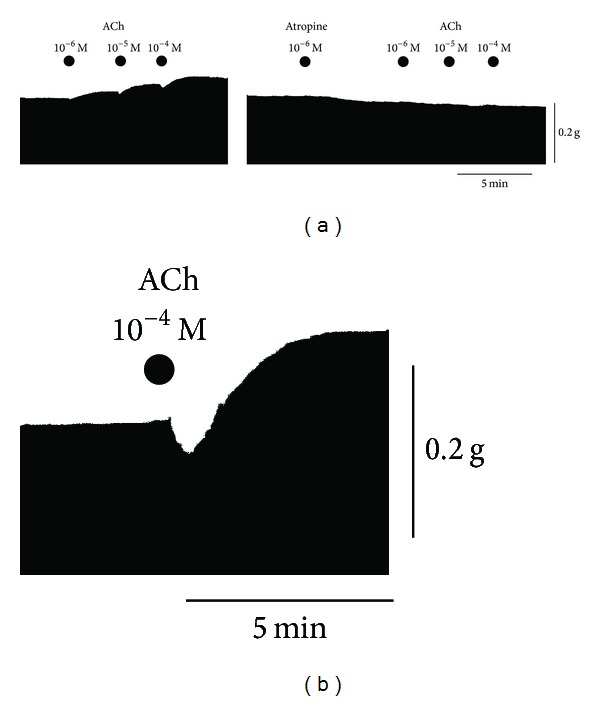
Inotropic effects of ACh on canine cardiac Purkinje fibers and effects of pretreatment with atropine on the ACh-evoked responses. (a) Typical tracings of effects of cumulatively applied ACh and atropine pretreatment on ACh-evoked inotropy. (b) Typical tracings of single application of ACh 10^−4^ M on the inotropy.

**Figure 2:**
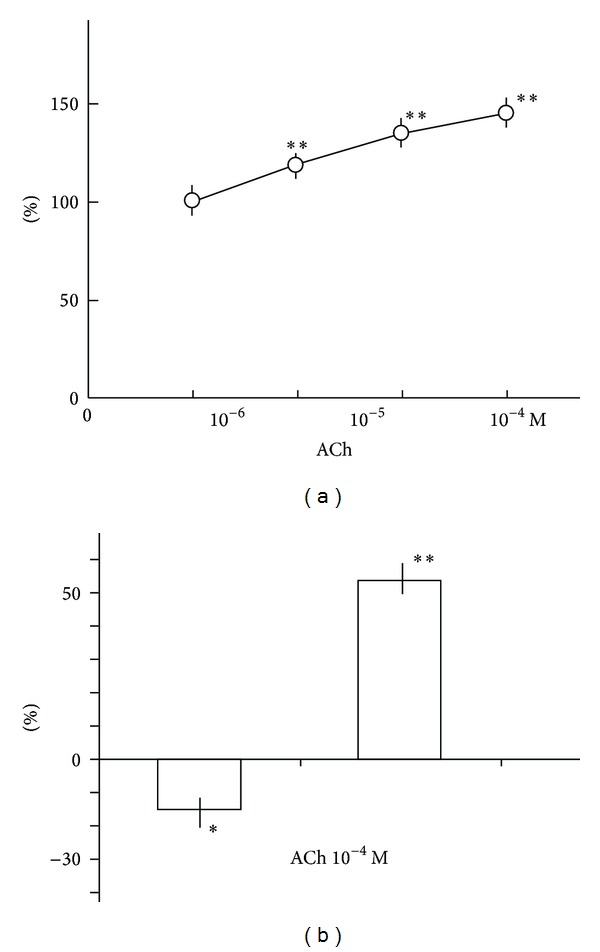
Effects of ACh on contractile force in canine cardiac Purkinje fibers. (a) Concentration-response relationships of the ACh-evoked positive inotropic effects in canine cardiac Purkinje fibers. Fibers were exposed in 1.8 mM Tyrode's solutions. Circles with vertical bars indicate the mean ± SEM (*n* = 5). (b) Biphasic responses of single application of ACh 10^−4^ M. Columns with vertical bars indicate the mean ± SEM (*n* = 4). Each point shows the rates of changes versus control, and statistical significance was performed using raw data. ∗∗ < 0.01, ∗ < 0.05 significantly different from control values ((a) one-way repeated measures ANOVA; (b) paired Student's *t*-test).

**Figure 3:**
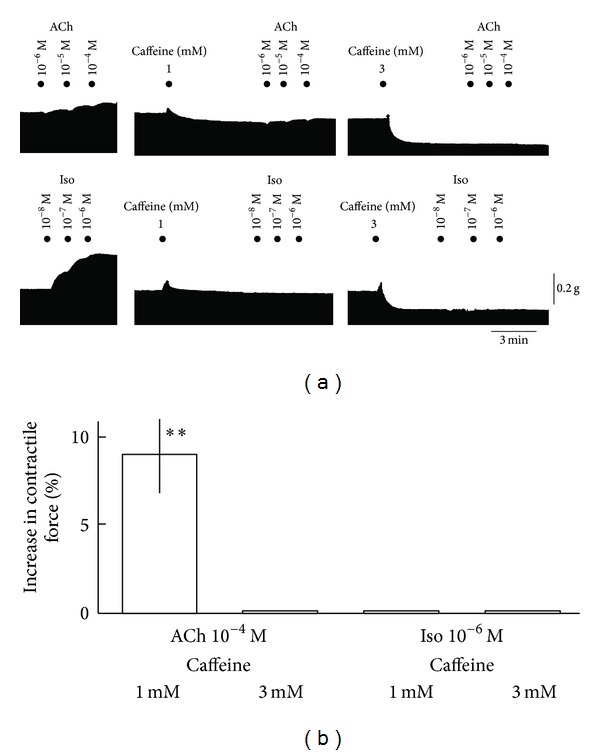
Influences of caffeine pretreatment on ACh- or Iso-evoked positive inotropy in canine cardiac Purkinje fibers. (a) Typical tracings of the inhibitory effects of caffeine on ACh-evoked inotropy or Iso-evoked inotropy. (b) Pooled data of effects of pretreated caffeine on ACh or Iso-evoked positive inotropy. Columns show the percent increase in contractile force in the presence of caffeine 1 or 3 mM, and vertical bar indicates SEM (*n* = 4). ∗∗ < 0.01 significantly different from control values before ACh or Iso application (paired Student's *t*-test or Wilcoxon's *t*-test).

Canine Purkinje fiber bundles were driven at 1 Hz.  Figure 1(a) shows that cumulatively applied acetylcholine (ACh) at concentrations of 10^−6^, 10^−5^, and 10^−4^ M decreased the contractile force transiently and then increased the contractile force in a concentration-dependent manner. In the experiment examining the effects of cumulatively applied ACh, the first application of ACh at 10^−6^ M decreased contractile force by 3.4 ± 0.5% (*P* < 0.01) and increased the force by 18 ± 3.3% (*P* < 0.01) (*n* = 5). In the experiment for a single application of ACh 10^−4^ M, the transient decrease in the contractile force was more evident, which was replaced by the increase in the contractile force (Figure 1(b)). These biphasic responses were abolished by the pretreatment with nonspecific antagonist, atropine 10^−6^ M completely.  Figure 2(a) shows concentration-response relationships of the positive inotropic actions caused by ACh.  Figure 2(b) shows pooled data of negative and positive inotropic effects provoked by single application of ACh 10^−4^ M. As shown in Figure 3(a), caffeine at 1–3 mM affected ACh- and Isoproterenol- (Iso-) induced changes of contractile force. The typical tracings of Figure 3(a) show that the Iso-induced increase in contractile force were affected more readily by the pretreatment with caffeine than ACh-induced one. The ACh (10^−4^ M)-evoked positive inotropy was attenuated by caffeine 1 mM but still remained a little (Figure 3(b)). On the other hand, the Iso (10^−6^ M)-evoked positive inotropy was inhibited completely by 100% by 1 mM caffeine. Caffeine 3 mM abolished ACh-evoked positive inotropy completely by 100% (Figure 3(b)). 

### 3.2. IP_3_ Levels in Canine Cardiac Purkinje Fiber Bundle Preparation

**Figure 4:**
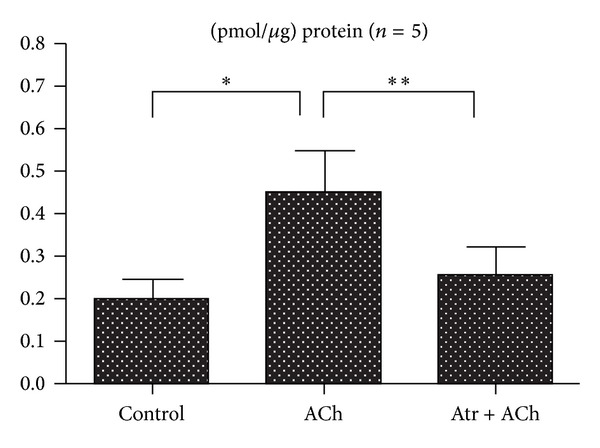
IP_3_ levels in canine cardiac Purkinje fibers treated by ACh or ACh and atropine (Atr). Columns with vertical bars indicate the mean ± SEM (*n* = 5). ∗ < 0.05, ∗∗ < 0.01 significantly different from each other (paired Student's *t*-test).

Results of IP_3_ levels in the samples are represented in Figure 4. IP_3_ levels in the Purkinje fibers incubated with ACh 10^−4^ M were significantly increased compared with the control. The ACh-evoked increase in IP_3_ levels was inhibited significantly to the control level by pretreatment with atropine 10^−5^ M. 

### 3.3. RT-PCR Products from the Canine Cardiac Purkinje Fibers, Atrial Muscle, and Salivary Gland

**Figure 5:**
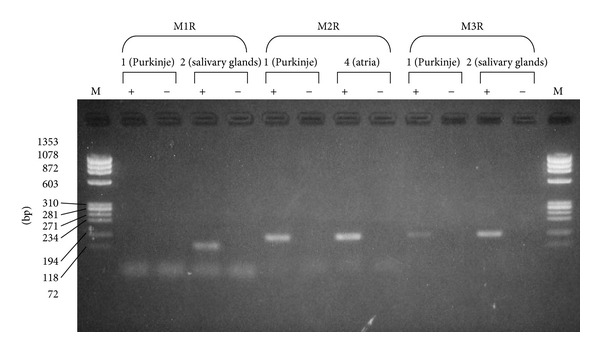
Gene expression of mAChR subtypes in Purkinje fibers. RT-PCR products for M_1_ (70 bp), M_2_ (106 bp), and M_3_ (109 bp) subtypes were analyzed by agarose gel electrophoresis. RT+ represents reverse-transcribed samples, and RT− represents corresponding reverse transcriptase-free controls. Salivary gland was used as positive control.

Expression of mAChR subtypes in Purkinje fibers was examined by RT-PCR (Figure 5). The RT-PCR product corresponding to M_2_ receptors was clearly detected in Purkinje fibers. The product corresponding to M_3_ receptors was also detected, but the amplitude of the expression was smaller than that of M_2_ receptors. Collected data showed about one fiftieth of the density of M_3_ receptor mRNA to that of M_2_ receptor mRNA (Table 1). M_1_ receptor expression was rarely detected. The products for all the three subtypes were clearly detected in positive control samples (salivary glands for M_1_ and M_3_ receptors and atrial muscles for M_2_ receptors). Genomic contamination was negligible because no PCR product was detected in any RT-free sample under the cycling conditions employed here. 

## 4. Discussion

M_2_ receptors are widely known to play important roles in the regulation of heart function. In addition, recent studies have demonstrated the existence of M_1_ and M_3_ receptors in the atria and ventricles, although the predominant subtype is M_2_ in the atria [2, 10]. M_3_ receptors were found in the heart of several species including humans, dogs, cats, guinea-pigs, rats, mice, and chicks. On the other hand, evidence against the presence of the M_3_ receptors has also obtained from some of these species [2]. M_2_ receptor stimulation induces negative inotropic and chronotropic actions by activating the G protein-coupled potassium channel and/or inhibiting adenylate cyclase [2, 11]. In contrast, the stimulation of M_3_ receptors was known to increase IP_3_ levels in the atria and ventricles [2, 3, 12]. There have been several electrophysiological or mechanical tension experiments showing the existence of muscarinic receptors by using pharmacological agents mainly such as M_1_ and M_2_ receptor antagonists in the cardiac Purkinje fibers. As a matter of fact, none of these studies came to the conclusion that the direct stimulation of muscarinic 3 receptor elicited the positive inotropy in cardiac Purkinje fibers. Curiously enough, muscarinic 3 receptor has not been paid much attention until now in the cardiac Purkinje fibers. M_1_ was related to positive inotropy or the increase in automaticity of the fibers, and M_2_ was related to negative inotropy or the decrease in the automaticity [5–8, 13].

In the studies using atria and ventricles, several studies demonstrated that M_3_ receptor stimulation increased prostaglandin (PG) production in the endocardial endothelium which caused positive inotropy in the mouse atria [14–17]. In contrast, Ateş and Kaygisiz reported that the positive inotropy was not dependent on PG production in rat hearts [18]. However, it is not evident whether PG is related to the positive inotropy or not in canine Purkinje fibers since we have not examined PG production in the present study.

The treatment with caffeine markedly affected the inotropy evoked by ACh. Caffeine was demonstrated to affect the positive inotropy by depleting the caffeine-sensitive Ca storage sites in the ventricular cells or tissues [19, 20]; thus, such actions were assumed to be exerted by caffeine in the Purkinje fibers as well. In the present study, the ACh-evoked positive inotropy was attenuated or abolished by the pretreatment with caffeine. The Iso-evoked positive inotropy was more readily affected by caffeine treatment. 1 mM caffeine was enough to abolish the positive inotropy caused by Iso at 10^−6^ M completely. Whereas ACh-evoked positive inotropy remained to a little extent after the treatment caffeine 1 mM, and 3 mM was needed to abolish the ACh-evoked positive inotropy completely. If ACh-evoked positive inotropy might be induced at least partly by IP_3_R-sensitive Ca release from Ca storage sites, and these sites are less sensitive to caffeine compared with ryanodine-sensitive Ca storage sites, then the lesser extent of the inhibition of ACh-evoked positive inotropy by the treatment with caffeine would be plausible [21]. It is known that open probability of the ryanodine receptors (RyRs), mainly responsible for contraction, is increased resulting from a local increase of cytoplasmic Ca^2+^ concentration. It might be possibly occurred that the increase in local [Ca^2+^]_i_ near vicinity of RyRs is subsequently induced by IP_3_R-induced Ca^2+^ release in canine cardiac Purkinje cells as indicated by Stuyvers et al. [21, 22]. It was demonstrated that millimolar concentrations of caffeine blocks IP_3_Rs [23], thus caffeine 3 mM eventually abolished ACh-evoked inotropy by decreasing the Ca^2+^ contents of IP_3_-sensitive and RyR-sensitive Ca stores to the subthreshold levels for contraction. As a matter of fact, recently there is great and growing interest in cardiac IP_3_ signaling on intracellular Ca^2+^ movements in various cardiac cells. Stuyvers et al. demonstrated the existence of IP_3_ receptors in canine Purkinje fibers by using the immunolabeling study [21]. They also demonstrated that IP_3_Rs and related local Ca^2+^ release have been observed near the sarcolemmal membrane. These phenomena might constitute the primary event of the sequence leading to large Ca^2+^ transients and subsequent cell wide Ca^2+^ waves, which they assume to possibly leads to the positive inotropy in canine Purkinje fibers [21, 24–28]. Taken together various experimental results regarding Ca^2+^ movements, and the results obtained from the present study, it is highly speculated that the activation of IP_3_Rs may be involved in ACh-provoked positive inotropy in canine Purkinje fibers. The ACh-induce activation of IP_3_Rs might be related with pathological states such as arrhythmias and heart failure [29].

It is concluded that canine cardiac Purkinje fibers have M_3_ receptors as well as M_2_ receptors. The density of M_3_ receptors are smaller than the density of M_2_ receptors, however, the physiological function was larger than expected from M_3_ receptor density. M_1_ receptors rarely exist, which differs from the other authors' findings. The present study demonstrated that the activation of M_3_ receptors caused by ACh augments the contractile force possibly by increasing IP_3_ levels. However, the significance of the functional role of M_3_ receptors still remains to be elucidated.

## Figures and Tables

**Table 1:** Amount of gene expression of mAChR subtypes in Purkinje fibers.

	M_1_	M_2_	M_3_
Purkinje fiber	<6.0 × 10^−4^	1.6 × 10^−1^ ± 8.5 × 10^−3^	1.5 × 10^−3^ ± 1.2 × 10^−4^
Salivary gland	5.1 × 10^−2^ ± 2.3 × 10^−2^	3.9 × 10^−3^ ± 1.6 × 10^−3^	1.5 × 10^−2^ ± 6.3 × 10^−3^
Atrial muscle	<4.4 × 10^−4^	2.1 × 10^−1^ ± 1.1 × 10^−2^	8.6 × 10^−4^ ± 4.1 × 10^−4^
